# Detachable coils and glue embolization for hemoptysis secondary to anomalous systemic arterial supply to the left lower lobe: a case report and literature review

**DOI:** 10.3389/fmed.2025.1627401

**Published:** 2025-08-11

**Authors:** Congqing Xie, Gaoyi Li, Wei Wei, Weiming Wang, Bin Yang, Xun Zhang, Wei Hu, Yanneng Xu, Guangyan Si, Gang Yuan

**Affiliations:** ^1^Department of Intervention and Vascular, The Affiliated Traditional Chinese Medicine Hospital, Southwest Medical University, Luzhou, China; ^2^Department of Respiratory Medicine, Luxian People’s Hospital, Luzhou, Sichuan, China; ^3^Department of General Surgery (Thoracic Surgery), Hejiang People’s Hospital, Luzhou, Sichuan, China; ^4^Department of Thoracic Surgery, The People's Hospital of Longmatan District, Luzhou, Sichuan, China; ^5^Department of General Surgery (Vascular Surgery), The Affiliated Hospital of Southwest Medical University, Luzhou, China; ^6^Department of Radiology, The Affiliated Traditional Chinese Medicine Hospital, Southwest Medical University, Luzhou, China

**Keywords:** anomalous systemic artery to left lower lobe, detachable coil, tissue glue, transcatheter arterial embolization, surgical operation

## Abstract

Anomalous systemic arterial supply to the left lower lobe (ASALLL) is a rare congenital vascular malformation. It predominantly occurs in young and middle-aged adults, often presenting with recurrent hemoptysis of unknown etiology. The condition has a high clinical misdiagnosis rate, and its optimal treatment remains controversial. Traditional management involves surgical or thoracoscopic lobectomy with ligation of the anomalous artery. In recent years, with advancements in endovascular techniques, transcatheter arterial embolization (TAE) has gained increasing preference among patients due to its minimally invasive and highly effective nature. Previous cases have primarily employed coils or vascular plugs to occlude the anomalous artery, with no reported use of detachable coil combined with medical tissue glue for embolization. Here, we present the case of an 18-year-old male with hemoptysis, diagnosed with ASALLL via CTA, who underwent successful embolization of the anomalous systemic artery using detachable coils combined with medical tissue glue. The procedure was uneventful, with excellent postoperative recovery. At the 1-year follow-up, the patient remained asymptomatic, with no complications such as pulmonary necrosis or infection. We also reviewed recent literature on the diagnosis and management of this condition, discussing key points in its diagnosis and differential diagnosis, and comparing the advantages and disadvantages of surgical intervention versus TAE. It aims to provide more robust evidence for the accurate diagnosis and optimal treatment selection of this rare vascular anomaly.

## 1 Introduction

Anomalous systemic arterial supply to the left lower lobe (ASALLL) refers to a rare congenital vascular malformation in which an aberrant systemic artery originating from the thoracic aorta supplies the basilar segments of the left lower lobe, where the bronchial and pulmonary parenchymal development is normal, with subsequent venous drainage into the left atrium via the inferior pulmonary vein (1, 2). On computed tomography (CT), a markedly enlarged aberrant artery supplying the left lower lobe can be observed, often leading to misdiagnosis as pulmonary sequestration (PS) or pulmonary arteriovenous fistula (PAVF) (3). The exact etiology remains unclear but may be related to abnormal regression of primitive aortic branches supplying the embryonic lung bud during development. Due to the plexiform lesions of primitive pulmonary arterial branches during embryogenesis, the primitive aortic branches fail to regress and continue to supply the lung bud. Consequently, the basal segmental pulmonary artery of the affected lower lobe is typically absent (4). Patients may remain asymptomatic, but some present with hemoptysis and/or chest discomfort secondary to pulmonary hypertension, often triggered by physical exertion. In rare cases, prolonged left-to-left shunting may lead to heart failure and respiratory distress (5).

Current treatment options include endovascular therapy and surgical interventions such as arterial ligation, segmentectomy, lobectomy, or anastomosis with the native pulmonary artery (1). However, there is no consensus on the optimal treatment strategy for ASALLL. Notably, since the anomalous artery arises directly from the aorta and is exposed to systemic arterial pressure, surgical dissection carries a high risk of massive hemorrhage. To minimize surgical risks and reduce trauma, transcatheter arterial embolization (TAE) of the aberrant artery has been reported with favorable outcomes (6). Herein, we present a case of ASALLL successfully treated with TAE using detachable coils combined with medical glue, achieving excellent clinical results. At the 1-year follow-up, no recurrence or major complications were observed.

## 2 Case presentation

**Figure 1:**
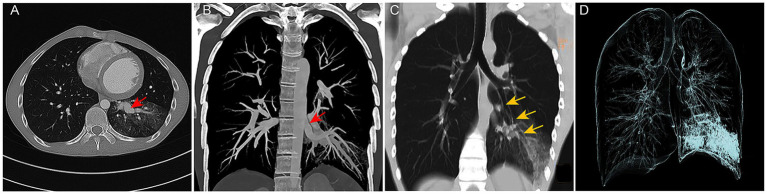
CT imaging findings of the patient’s chest. **(A)** On CT lung window settings, patchy hazy opacities are visible in the left lower lobe, with abnormally dilated and tortuous vessels forming a localized soft tissue nodule (indicated by red arrow). **(B)** A markedly enlarged anomalous artery originating from the distal thoracic aorta is observed coursing through the left lower lobe parenchyma (red arrows), accompanied by patchy consolidation and ground-glass opacities in the left lower lobe. **(C,D)** CT coronal reconstruction and 3D bronchial tree rendering demonstrate normal continuity between the left lower lobe bronchus and the native bronchial tree (yellow arrows).

An 18-year-old male was admitted to the respiratory department due to “intermittent cough and hemoptysis for one day.” One day before admission, the patient developed a cough accompanied by hemoptysis (approximately 10 mL) after physical exertion, which resolved with rest. However, subsequently, even minor activity triggered coughing and hemoptysis, with a cumulative daily volume of nearly 100 mL, along with a sensation of a foreign body in the throat. He did not experience chills, high fever, dyspnea, or other discomforts. His past medical history was unremarkable except for rickets. Physical examination revealed coarse breath sounds and moist rales in the left lower lung. Laboratory tests showed no significant abnormalities. However, initial CT demonstrated hazy opacities in the left lower lobe, suggestive of pulmonary infection with alveolar hemorrhage. Additionally, abnormally dilated vessels and a localized soft tissue nodule (Figure 1A) raised suspicion of a vascular malformation. Consequently, a chest CTA was performed, revealing a large aberrant artery (maximum diameter: 1.2 cm) originating from the distal thoracic aorta and coursing through the left lower lobe, accompanied by patchy consolidation and ground-glass opacities (Figure 1B). The affected lung tissue maintained normal architecture and bronchial connectivity (Figures 1C,D). After multidisciplinary consultation involving radiology, interventional vascular surgery, and thoracic surgery, the patient was diagnosed with ASALLL. Following detailed informed consent, he opted for endovascular treatment.

**Figure 2:**
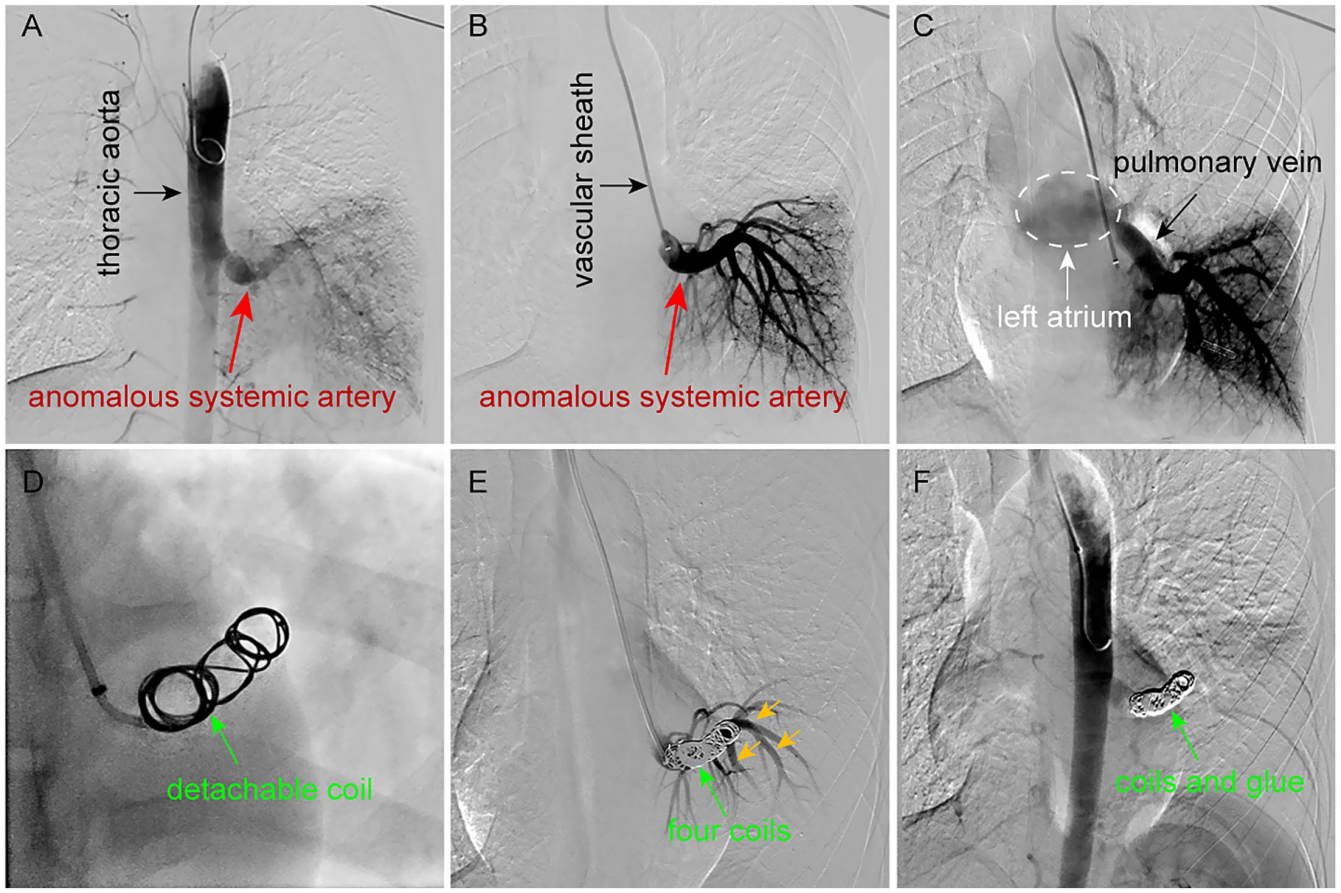
Endovascular embolization procedure for ASALLL. **(A,B)** DSA reveals an abnormally enlarged branch vessel (diameter: 1.2 cm) arising from the descending aorta, which directly supplies the left lower lobe parenchyma. **(C)** The anomalous systemic artery ultimately drains into the left atrium via pulmonary venous return. **(D)** A 12-mm detachable coil was deployed in the main trunk of the aberrant artery. **(E)** Post-embolization DSA after placement of four coils demonstrates incomplete occlusion, with residual filling of branch vessels (yellow arrows). **(F)** Supplemental injection of medical glue (IBCA) through the coil mesh achieves complete embolization, with no further contrast opacification of either the main trunk or its branches.

The procedure was performed under local anesthesia via a left brachial artery approach. Digital subtraction angiography (DSA) confirmed the anomalous artery arising from the descending aorta and draining into the left atrium via the pulmonary vein (Figures 2A−C). A 6-French long sheath (Cook Medical, United States) was positioned at the origin of the aberrant artery to facilitate subsequent interventions. Two Interlock coils (12 mm diameter, Boston Scientific, MA, United States) and two conventional coils (10 mm diameter, Cook Medical, United States) were deployed to embolize the main trunk (Figures 2D,E). However, due to the vessel’s large caliber, post-embolization DSA revealed incomplete occlusion. To enhance embolization efficacy and avoid excessive metal coil implantation, a 2.6-French microcatheter (Stride, Asahi Intecc Co., Ltd., Aichi, Japan) was advanced through the coil interstices under wire guidance. A mixture of medical glue (Isobutyl-2-cyanoacrylate, IBCA, TycoBerman Medical Technologies Co., Ltd. Hubei, China) and lipiodol (1:1 ratio) was then slowly injected (0.5 mL per minute, and the total amount is about 1 mL) to supplement the embolization. The glue was distributed optimally without non-target embolization. Final DSA confirmed complete occlusion, with coils and glue firmly anchored in the vessel lumen (Figure 2F). The patient remained asymptomatic during the procedure, and hemoptysis ceased immediately postoperatively.

**Figure 3:**
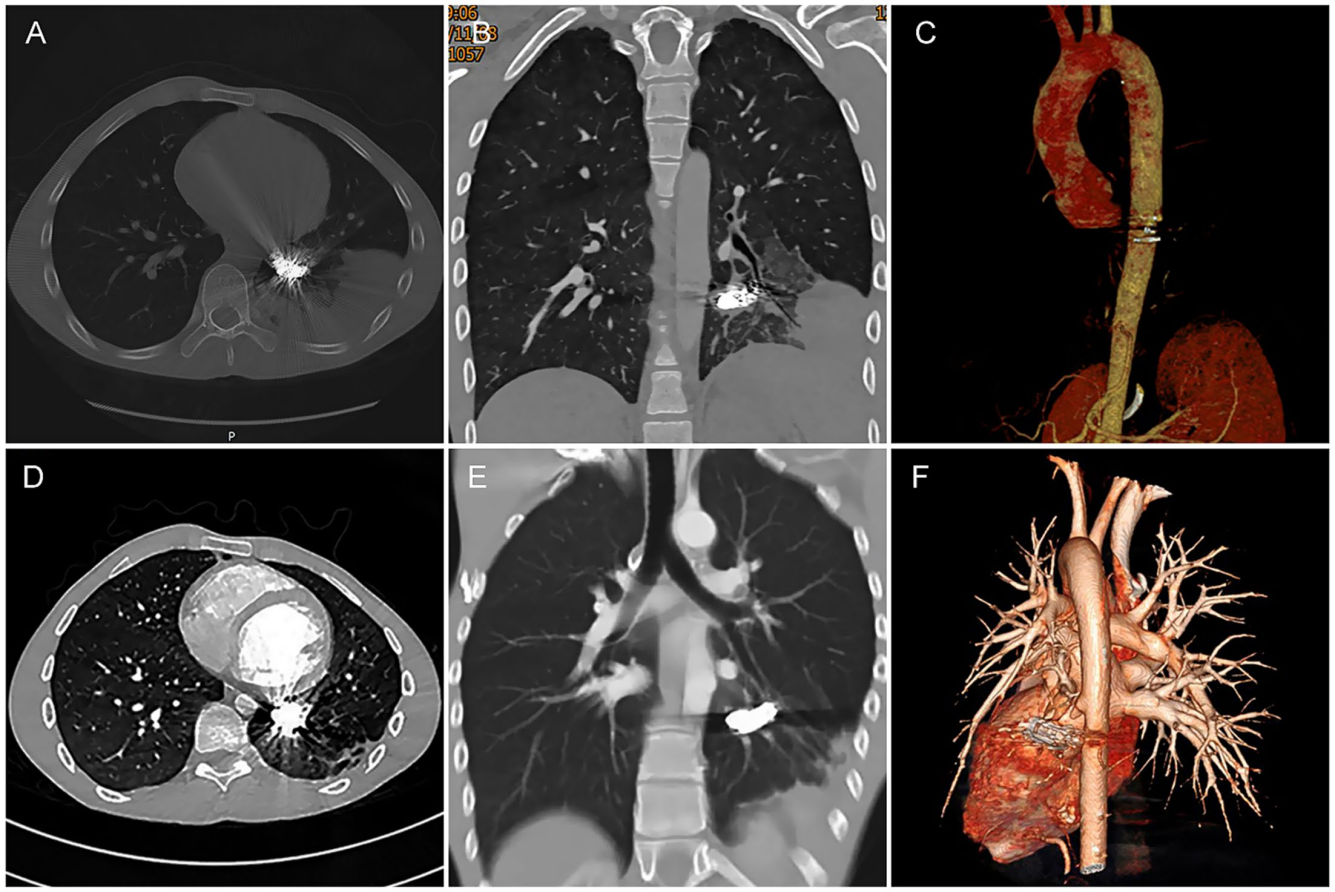
Post-embolization CT follow-up. **(A−C)** Three-day post-TAE CTA demonstrates: Embolic materials (coils and glue) firmly positioned within the lumen of the occluded anomalous artery. A mass-like high-density shadow was observed in the left lower lobe, surrounded by multiple patchy consolidations and ground-glass opacities. The previously abnormal artery and its branches were no longer visible, suggesting localized infarction of the left lower lobe following embolization. **(D−F)** One-year follow-up CTA reveals: Only minor patchy and linear opacities were noted in the left lower lobe. Mild reduction in left thoracic volume (parenchymal collapse) was observed; no significant pleural effusion, pulmonary abscess, or tissue necrosis was detected. The original aberrant systemic artery and its tributaries remained persistently invisible, indicating favorable post-embolization outcomes.

To mitigate post-embolization local inflammatory reactions and prevent pulmonary infection, the patient received intravenous dexamethasone sodium phosphate 10 mg daily for 3 days postoperatively, along with oral cefixime dispersible tablets 100 mg twice daily. Follow-up CT at 3 days revealed a mass-like high-density shadow in the left lower lobe, surrounded by multiple patchy consolidations and ground-glass opacities (Figures 3A−C), attributed to post-procedural localized infarction and inflammatory changes rather than infection or abscess formation. He was discharged after completing 5 more days of anti-inflammatory and antibiotic therapy. At the 1-year follow-up, he remained asymptomatic, with CTA revealed complete occlusion of the previously abnormal blood vessels post-embolization. Furthermore, imaging revealed only minor patchy and linear opacities were noted in the left lower lobe, with no evidence of pulmonary infection, or tissue necrosis (Figures 3D−F).

## 3 Discussion

ASALLL is a rare congenital disorder, predominantly affecting the left lower lobe. Due to its overlapping clinical and imaging features with intralobar pulmonary sequestration (PS), it is frequently misdiagnosed as such. In fact, this condition was initially termed “Pryce type I sequestration” in 1946 by Pryce and later described by other scholars as “systemic arterial supply to normal basal segments of the left lower lobe without sequestration” or “anomalous systemic arterial supply to non-sequestered lung” (7, 8). However, with advancements in imaging technology and improved understanding of its anatomical basis, it has become clear that ASALLL is distinct from PS. Although the affected lung segment is supplied by an anomalous systemic artery, the lung tissue itself is not sequestered, and the bronchial tree develops normally. In contrast, PS arises during embryogenesis when a portion of lung tissue separates from the normal tracheobronchial tree and develops independently. This sequestered tissue is not only perfused by an aberrant systemic artery but also forms isolated bronchial structures that do not communicate with the normal airways and lack respiratory function (9).

These anatomical differences also lead to distinct clinical manifestations. In PS, the sequestered lung tissue lacks communication with the normal bronchial tree, making it more prone to recurrent pulmonary infections. The aberrant systemic arteries supplying PS are typically smaller in caliber, resulting in fewer intrapulmonary hemorrhages. Even when bleeding occurs, the absence of bronchial drainage prevents hemoptysis, so patients rarely present with this symptom. In contrast, ASALLL involves normally developed lung tissue with intact bronchial connections, reducing susceptibility to infection. However, the aberrant systemic artery in ASALLL is usually larger in diameter, delivering a high blood flow to the basal segments of the left lower lobe. This leads to pulmonary vascular congestion and a higher likelihood of alveolar hemorrhage, often manifesting as significant hemoptysis (5). Thus, while both ASALLL and PS involve abnormal pulmonary vascular supply, they differ significantly in embryological origin, pathological mechanisms, and clinical characteristics. To facilitate accurate differentiation, we summarize the key diagnostic distinctions between these two entities in Table 1.

**Table 1:** Key points for distinguishing ASALLL from PS.

Feature	ASALLL	PS
Isolated lung tissue	No (connected to bronchial tree)	Yes (not connected to bronchial tree)
Pathological basis	Abnormal blood supply only	Lung maldevelopment, and abnormal blood supply
Venous drainage	Typically to pulmonary veins (left atrium)	Intralobar: pulmonary veins; Extralobar: systemic veins
Complications	Hemoptysis, heart failure	Intralobar: infection; Extralobar: usually asymptomatic
Associated anomalies	Rare	Common in extralobar type (e.g., diaphragmatic hernia, congenital heart disease)
Imaging key findings	Abnormal artery, and normal lung architecture	Abnormal artery, and non-ventilated lung (cystic or solid mass)

In addition to PS, this condition must also be differentiated from PAVF, which is a vascular complex composed of feeding arteries, nidus, and draining veins, characterized by direct communication between pulmonary arteries and veins without an intervening capillary network. Approximately 95% of PAVF feeding arteries originate from the pulmonary artery, while only 5% arise from systemic arteries (10). Accurate diagnosis relies on contrast-enhanced CT, which typically demonstrates linear or nodular high-density shadows beneath the pleura. During the arterial phase, these lesions exhibit rapid and marked enhancement, with an intensity similar to adjacent vessels. Three-dimensional vascular reconstruction further reveals the lesion’s connection to the feeding artery at one end and the draining pulmonary vein at the other.

Hemoptysis is often the initial symptom of ASALLL. Without timely intervention, persistent pulmonary venous hypertension can lead to left atrial dilation, congestive heart failure, cough, exertional wheezing, chest pain, and even dyspnea. Therefore, prompt therapy is necessary for symptomatic patients to prevent progression to cardiopulmonary failure. Currently, both surgical resection and TAE are viable treatments for ASALLL. However, due to differences in efficacy and safety, the optimal treatment strategy remains controversial.

Surgical management typically involves ligation of the anomalous systemic artery and resection of the affected lobe or segment. With advancements in surgical techniques and instrumentation, the approach has evolved from open thoracotomy with lobectomy to minimally invasive options such as Video-assisted thoracoscopic surgery (VATS) lobectomy, anatomic segmentectomy, and thoracoscopic anastomosis of the anomalous systemic artery to the pulmonary artery (11, 12). However, in adult patients, the long-term exposure to high-pressure systemic arterial flow leads to vascular wall thickening and reduced elasticity, resulting in low perfusion rates after anastomosis. Consequently, some experts suggest that vascular anastomosis is less suitable for adults and may be more appropriate for pediatric patients, who typically have a shorter disease duration and better vascular adaptability (13).

Surgical treatment offers distinct advantages in correcting anatomical abnormalities and preventing recurrence, yet its adoption is limited by significant trauma and uncontrolled intraoperative bleeding risks. Minimally invasive TAE has thus emerged as an effective alternative. As early as 1998, Bruhlmann et al. (14) achieved successful ASALLL treatment via coil embolization of anomalous systemic arteries. Recent studies further confirm TAE’s short-term efficacy, with its minimally invasive profile proving particularly valuable for high-risk populations like pregnant women and adolescents (6, 15, 16). However, long-term outcomes remain uncertain due to limited follow-up data, and concerns persist regarding recurrence in cases with large anomalous arteries or incomplete embolization. A hybrid approach, preoperative embolization followed by surgery, has shown promise in reducing bleeding risks while addressing recurrence concerns, synergizing both techniques’ benefits (17).

Currently, the majority of interventionalists still prefer using metal coils alone for embolizing the anomalous artery in ASALLL. Some scholars have proposed alternative approaches, such as the amplatzer vascular plug (AVP) for endovascular treatment of this condition (18). However, there are very few reported cases utilizing detachable coils for ASALLL, and even fewer documenting the combined application of detachable coils with medical glue (e.g., IBCA). AVP is a self-expanding cylindrical mesh device primarily used for treating atrial septal defects and patent ductus arteriosus. Selecting an appropriately sized AVP can also be used for the embolization of the abnormal systemic artery. Although a small number of case reports have documented its successful application in ASALLL therapy, it has not been widely adopted for ASALLL due to the limited number of cases and its high cost. In contrast, the detachable coils offer significant advantages, including adjustable deployment and optimal packing density during embolization procedures. This controllability allows for precise repositioning when initial placement is suboptimal, enabling operators to achieve satisfactory occlusion through iterative adjustments during the intervention. Medical glue is a cost-effective and easy-to-use liquid embolic agent with superior properties. When combined with detachable coils, it not only enhances embolization efficiency but also reduces the patient’s medical burden. In this case, the patient’s pathological vessel exhibited a large caliber that would have required dozens of conventional metal coils to achieve a complete embolization endpoint. Such an approach would have substantially increased the medical burden on the patient. Instead, we implemented an optimized strategy employing detachable coils in combination with ordinary coils, successfully occluding the majority of blood flow using merely four devices. This foundational embolization was then supplemented with a minimal quantity of tissue glue, resulting in complete and durable vascular occlusion. Even during the follow-up 1 year later, CTA showed no recanalization of the patient’s diseased blood vessels or change in the position of the embolic agent, and the patient did not experience adverse reactions such as lung infection and chest pain. This approach demonstrates that the use of detachable coils with adjunctive glue is a viable, efficient, and economically favorable strategy for managing large-caliber ASALLL vessels.

## 4 Conclusion

In summary, ASALLL is a clinically rare entity that is prone to misdiagnosis. Clinicians should maintain a high index of suspicion and carefully differentiate it from PS and PAVF. For patients presenting with unexplained hemoptysis, particularly young adults, prompt CTA should be performed to establish a definitive diagnosis. Both surgical intervention and TAE have distinct advantages and limitations. Treatment selection should be tailored to individual patient characteristics to ensure optimal safety and efficacy. TAE is a safe and effective approach for ASALLL. The combined use of detachable coils and medical glue not only enhances embolization efficiency but also reduces the economic burden on patients. This technique demonstrates significant clinical value and warrants broader adoption in clinical practice.

## Data Availability

The original contributions presented in the study are included in the article/supplementary material, further inquiries can be directed to the corresponding authors.
